# Mixed Viral-Bacterial Infections and Their Effects on Gut Microbiota and Clinical Illnesses in Children

**DOI:** 10.1038/s41598-018-37162-w

**Published:** 2019-01-29

**Authors:** Shilu Mathew, Maria K. Smatti, Khalid Al Ansari, Gheyath K. Nasrallah, Asmaa A. Al Thani, Hadi M. Yassine

**Affiliations:** 1 Biomedical Research Center, Qatar University, Doha, 2713 Qatar; 2 Pediatric Emergency Center, Hamad Medical Corporation, Doha, 3050 Qatar; 3 Department of Biomedical Science, College of Health Science, Qatar University, Doha, 2713 Qatar

## Abstract

Acute gastroenteritis remains a major cause of morbidity and mortality among young children worldwide. It accounts for approximately 1.34 million deaths annually in children younger than five years. Infection can be caused by viral, bacterial and/or parasitic microorganisms. Dysbiosis due to such infections could dramatically affect disease prognosis as well as development of chronic illness. The aim of this study was to analyze gut microbiome and clinical outcomes in young children suffering from viral or mixed viral-bacterial infection. We evaluated gut microbiota composition in children suffering from viral or mixed viral-bacterial infection with two major viruses rotavirus (RV) and norovirus (NoV) and two pathogenic bacteria [*Enteroaggregative E*. *coli* (EAEC), and *Enteropathogenic E*. *coli* (EPEC)]. We sequenced 16S ribosomal RNA (V4 region) genes using Illumina MiSeq in 70 hospitalized children suffering from gastroenteric infections plus nine healthy controls. The study summarized Operational Taxonomic Unit (OTU) abundances with the Bray-Curtis index and performed a non-metric multidimensional scaling analysis to visualize microbiome similarities. We used a permutational multivariate analyses of variance to test the significance of group differences. We also analyzed the correlation between microbiome changes and clinical outcomes. Our data demonstrated a significant increase in the severity score in children with viral-bacterial mixed infections compared to those with virus infections alone. Statistical analysis by overall relative abundance denoted lesser proportions of *Bacteroides* in the infected children, whereas *Bifidobacteriaceae* richness was more prominent in the bacterial-viral mixed infections. Pairwise differences of gut microbiota were significantly higher in RV + EAEC (P = 0.009) and NoV + EAEC (P = 0.009) co-infections, compared to EPEC mixed infection with both, RV (P = 0.045) and NoV (P = 0.188). Shannon diversity index showed considerable more variation in microbiome diversity in children infected with RV cohort compared to NoV cohort. Our results highlight that richness of *Bifidobacteriaceae*, which acts as probiotics, increased with the severity of the viral-bacterial mixed infections. As expected, significant reduction of relative numbers of *Bacteroides* was characterized in both RV and NoV infections, with more reduction observed in co-infection pathogenic *E*. *coli*. Although mixed infection with EAEC resulted in significant microbiota differences compared to viral infection only or mixed infection with EPEC, the clinical condition of the children were worsened with both pathogenic *E*.*coli* co-infections. Further, in comparison with RV cohort, augmented number of differential abundant pathogenic OTUs were peculiarly noticed only with NoV mixed infection.

## Introduction

The human gastrointestinal tract (GIT) is a reservoir of the largest community of commensals in the body, and hence, recent research has studied gut microbiome extensively^1,2^. Gut microflora plays a crucial role in health and disease through maintaining several physiological processes such as food digestion, immunity and metabolism^3–6^. Consequently, alteration of the structure and function of gut microflora has been linked to several human complications including colonization of pathogenic bacteria, susceptibility to autoimmune disorders, obesity, and gastrointestinal disorders such as inflammatory bowel disease^1,7–9^.

Healthy children gut microbiota is dynamic and undergoes rapid changes, which is affected by multiple factors including mode of birth delivery, aging process, diet, and use of antibiotics^10^. GIT microbiome in children is largely colonized by bacteria belonging to Bacteroidetes, Firmicutes and Proteobacteria phyla^11^. Facultative bacteria such as *Escherichia coli* (*E*. *coli*), *Enterococcus*, α-hemolytic *Streptococci*, and *Staphylococcus* species have been found to colonize the anaerobic GIT of infants during their first days after birth, followed by colonization of anaerobic bacteria including *Bacteroides*, *Bifidobacterium*, and *Clostridium* species, due to the presence of anaerobic conditions and human milk oligosaccharides^12^. Typically, the healthy gut microbiota is composed of only a minor proportion of Proteobacteria phylum, and thus, high abundance of these bacteria is often a sign of imbalanced microbiome^13,14^.

GIT is also a common site of infections in children younger than 5 years^15^. Acute gastroenteritis (AGE) leads to around 1.34 million deaths annually, or nearly 15% of all child fatalities^16,17^. Viral infections remain the leading cause of AGE in children, particularly noroviruses (NoV) and rotaviruses (RV)^18^. NoV are group of RNA viruses that are responsible for about one fifth of AGE cases globally^19^. Annually, these viruses lead to around 200 million cases in children less than 5 years old, and result in about 50,000 fatalities^19,20^. RV is also one of the main viral agents of AGE worldwide. Despite the availability of rotavirus vaccine (RVV), this virus is the most common cause of diarrhea related deaths in children, contributing to 215,000 deaths in young children every year, particularly in low-income countries^21,22^.

Gut microflora plays a critical role in immune response and pathogenesis of GIT infection especially in young children, where GIT infections are major cause of morbidity and mortality. Accordingly, several studies have investigated the correlation between intestinal microbiota composition and immune response to RVV^22–24^. On the other hand, immune tolerance to gut microflora is an essential component of mucosal immunity^25^. Loss of this immune tolerance has been reported as a consequence GIT infection, where immune response to commensals parallels the immune response to pathogenic microorganisms^25^. Virally-infected infant’s exhibit altered gut microbiota after infection, which may put them at higher risk of developing health complications^26^. More recently, it was reported that children with severe viral AGE, particularly patients infected with RV, have decreased intestinal microbiota diversity compared to healthy controls^24^. The use of antibiotics following viral infections has also been linked to gut microbiota alteration in children^25–27^. Nonetheless, the current data concerning the contribution of gut microbiota in the development, complications, and pathogenesis of AGE is still limited, which underscores the need for more investigations.

In this present study, we evaluated gut microbiota composition in children suffering from viral or mixed infection with two major viruses (RV and NoV) and two pathogenic bacteria [*Enteroaggregative E*. *coli* (EAEC), and *Enteropathogenic E*. *coli* (EPEC)]. In addition, we investigated the correlation between gut microbiome alterations due to various infections and disease manifestations.

## Results

### Patient characteristics and study design

A total of 79 fecal samples were analyzed, including 70 stool samples from children suffering from AGE [RV (n = 40) and NoV (n = 30); median age of 14 months], and nine samples from healthy children [median age of 13.5 months]. Of the 40 RV positive children, 18 children had RV-alone infection (Vesikari score ≤10), eight had mixed infection with RV + EAEC (Vesikari score >10), nine had RV + EPEC infection (Vesikari score > 10) and five samples had RV + EPEC + EAEC mixed infection (Vesikari score > 10). Of the 30 NoV positive samples, 17 had NoV-alone infection (Vesikari score ≤10), eight had NoV + EAEC (Vesikari score <10) and five children had NoV + EPEC infection (Vesikari score > 10). We did not have NoV + EPEC + EAEC, nor single bacterial infections to include in the analysis. The number of children vaccinated with RVV in the NoV group [NoV: 76.5% (13/17), NoV + EAEC: 71.4% (5/7), NoV + EPEC: 80% (4/5)] was higher compared to RV group [RV: 61.1% (11/18), RV + EAEC: 62.5% (5/8), RV + EPEC: 60% (6/10) and RV + EPEC + EAEC: 40% (2/5)]. Children admitted to pediatric emergency center with AGE were observed to have multiple clinical characteristics comprised of fever, prolonged period of diarrhea, vomiting, hospitalization and both mild to moderate dehydration. All fecal samples were collected before the administration of any medications. Table 1 summarizes the clinical characteristics and conditions of the children enrolled in this study.

**Table 1:** Clinical characteristics and conditions of the children enrolled in this study.

Clinical conditions	RV cohorts				NoV cohorts			Controls
	RV-alone	RV + EAEC	RV + EPEC	RV + EPEC + EAEC	NoV-alone	NoV + EAEC	NoV + EPEC	Healthy
Number of children	18	8	9	5	17	8	5	9
Number of males	11	6	7	2	12	5	4	5
Number of females	7	2	2	3	5	3	1	4
Median age of children (months)	19	28	29	15	15	9	11	13.5
Age range of children (months)	(3–72)	(11–84)	(5–72)	(10–24)	(4–60)	(4–23)	(7–108)	(11–84)
Diarrhea frequency (per day)	6–7	7–8	8–9	6–7	5–6	4–5	6–7	NA
Vomiting frequency (per day)	5–6	3–5	5–6	5–6	5–6	6–7	6–7	NA
Max reached fever (°C)	38.5	37.1	39	37.5	39.5	37.1	38.8	NA
Mean duration of hospitalization (days)	0–1	0–1	1–2	1–2	1	0–1	0–1	NA
Rotavirus vaccinated	61.1% (11/18)	62.5% (5/8)	60% (6/10)	40% (2/5)	76.5% (13/17)	71.4% (5/7)	80% (4/5)	NA
Degree of dehydration	Mild: 66.7%	Mild: 25%	Mild: 44.5%	Mild: 60%	Mild: 65%	Mild: 25%	Mild: 60%	NA
	Moderate: 33.3%	Moderate: 75%	Moderate: 55.5%	Moderate: 40%	Moderate: 35%	Moderate: 62.5%	Moderate: 40%	NA

NA: Not available.

### Mapping the microbiome community composition and biodiversity in AGE patients and healthy controls

**Figure 1:**
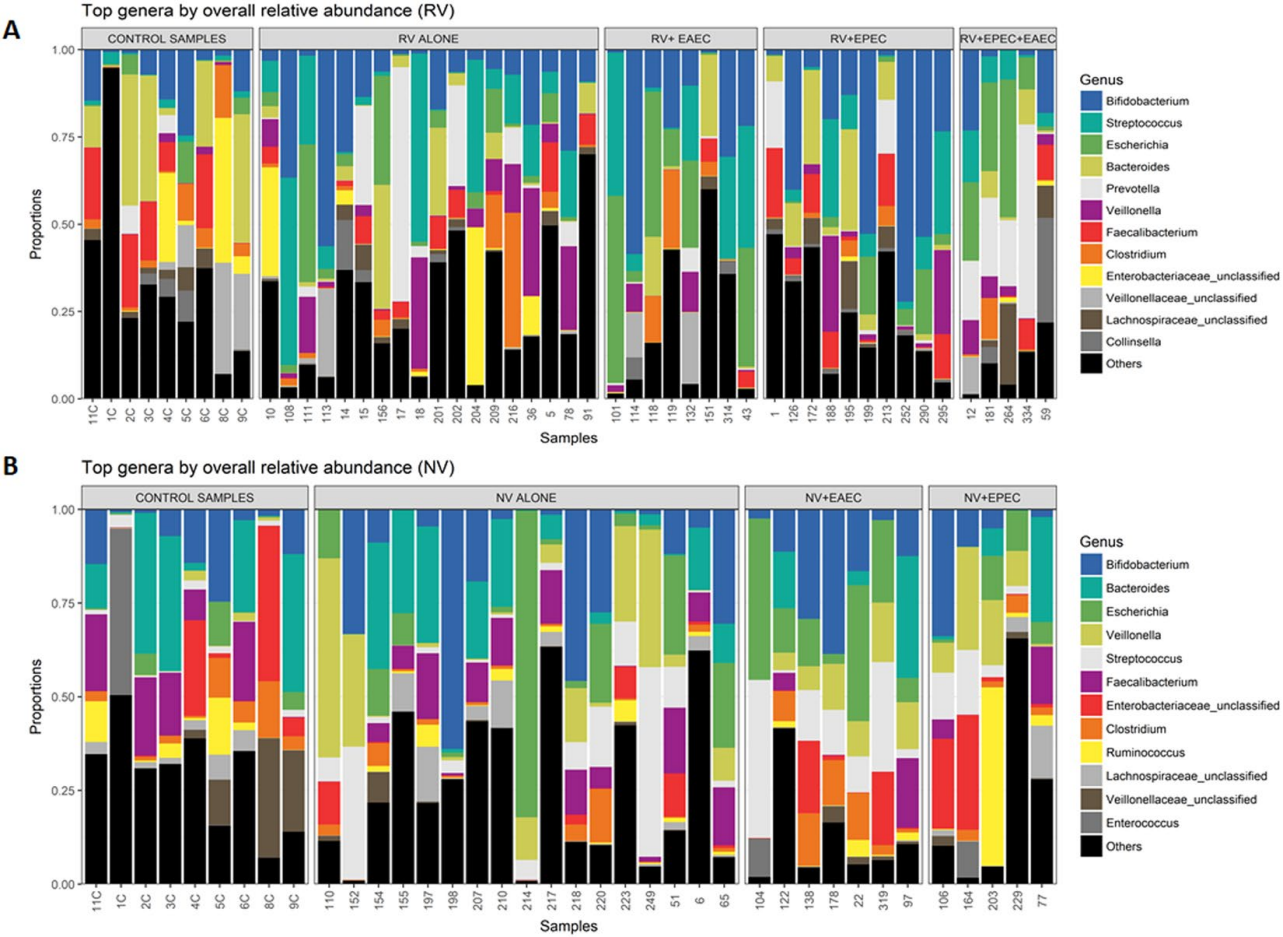
Community clustering and composition of top genera of bacterial species and their overall relative abundance in 79 children. All gut microbiota OTU profiles of the patients were aggregated into genera and plotted. “Other” under the genus represents lower-abundance taxa. Optimal number of consensus taxonomy was obtained for each OTU and 12 most abundant genera were displayed. (**A**) Clustering and composition of top 12 genera in RV cohort in comparison with control group. (**B**) Clustering and composition of top 12 genera in NoV cohort in comparison with control group.

We sequenced 16S (V4 region) genes on an Illumina MiSeq. Raw Fastq files were quality-filtered and clustered into 97% similarity operational taxonomic units (OTUs) using the mothur software package^28^. For all samples, we obtained 1.488075 × 10^6^ high-quality 16S rRNA sequence reads generated from Miseq Illumina platform. The final dataset yielded 8118 OTUs including those bacteria with a count of one. The read range which was between 1436 and 3.17 × 10^4^ denotes the size of the sequences aligned for the identified OTUs. High quality reads were classified using Greengenes (v. 13_8) as the reference database. We obtained a consensus taxonomy for each OTU. We then aggregated OTU abundances into genera, and plotted the relative abundances of the most abundant ones with Analysis of variance (ANOVA). We have represented a plot to denote the most abundant genera for each cohort (Fig. 1), as well as per each group independently (Supplementary Fig. 1). The results of positive correlation between relative abundance and prevalence was calculated by using Spearman’s correlation coefficient. We summarized OTU abundances with the Bray-Curtis index and performed a non-metric multidimensional scaling (NMDS) analysis to visualize microbiome similarities^28^. We used a Permutational analysis of variance (PERMANOVA) to test the significance of group differences^29^. Enteric bacteria in the 79 fecal samples were classified into 24 phyla, 154 families, and 273 genera based 16S rRNA sequences and metagenomics analysis of the selected reads. The complete AGE bacterial communities and the relative abundance for each genus, phylum and family level for all children samples are listed in Additional File 1. We categorized samples into two cohorts: RV cohort which includes RV, RV + EAEC, RV + EPEC, RV + EAEC + EPEC, and NoV cohort which includes NoV, NoV + EAEC, and NoV + EPEC. The relative abundance of species in each group was compared with other groups to determine the relatedness.

In all AGE samples, 273 bacterial genera were detected. Despite this large number of gut microbes, a relatively small collection of genera represented 90% of all bacterial reads in all samples. Distinct bacterial composition and communities were present between healthy samples as compared with RV and NoV groups, which differed in both diversity and composition [Fig. 1(A,B)]. We identified 12 genera showing significant differences of relative abundance in community between RV and NoV infected children. We found a positive correlation between relative abundance and prevalence of bacteria in both RV and NoV cohorts. That is, a genus highly abundant in one sample was also highly prevalent across all samples of the same group. The twelve most abundant genera in RV infected groups were *Bifidobacterium*, *Streptococcus*, *Escherichia*, *Bacteroides*, *Prevotella*, *Veillonella*, *Faecalibacterium*, *Clostridium*, *Collinsella* and unclassified genus of *Enterobacteriaceae*, *Veillonellaceae* and *Lachnospiraceae* [Fig. 1(A)]. Specifically, genera (abundance value) which includes *Streptococcus* (0.124), *Escherichia* (0.107), *Prevotella* (0.0759) and *Veillonella* (0.0633) showed the highest prevalence and relative abundance, and accounted in average for 77.5% of the bacteria in RV positive samples. In the RV + EAEC mixed-infection group, *Streptococcus* and *Escherichia* genera were also more prevalent and relatively abundant. Both genera declined in the RV + EPEC mixed-infection with a mean relative abundance of 0.25 and 0.37, though they were prevalent in all samples. Interestingly, predominant abundance of genera *Prevotella* and *Escherichia* was observed in RV + EAEC + EPEC mixed infections with a mean relative abundance of 0.75 when present. Relative abundance with respect to unclassified genera, *Lachnospiraceae* and *Collinsella*, were abundant in RV + EPEC and RV + EPEC + EAEC, while less abundant in RV + EAEC [Fig. 1(A)]. The twelve top bacterial genera and their overall relative abundance per each group individually are represented in Supplementary Data S1- Fig. 1. Abundance of specific genera was also noticed in each of RV groups, particularly *Oscillospira* (0.0141) (RV-alone and RV + EAEC), *Dialister* (0.0256) and *Megamonas* (0.0385) (RV + EPEC), and *Sutterella* (0.0122) (RV + EAEC + EPEC).

**Figure 2:**
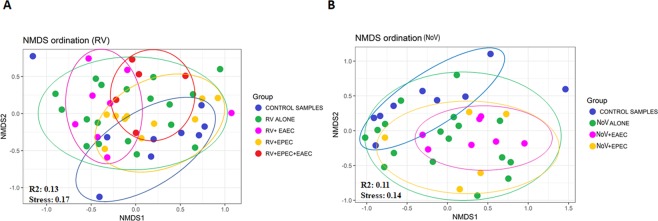
Nonmetric multidimensional scaling (NMDS) ordination of fecal microbiota in AGE infected patients. The Bray-Curtis index were performed between all infected children and controls to generate NMDS to visualize gut microbiome similarities. Each dot in the figure denotes microbiota profile of a single patient in a low-dimensional space. Colored dots denotes groups of patient’s microbiome. The subject’s cluster together depending upon their microbiome profiles. (**A**) NMDS ordination of microbiomes in control samples, RV-alone, RV + EAEC, RV + EPEC, and RV + EPEC + EAEC. (**B**) NMDS ordination of microbiomes in control samples, NV-alone, NV + EAEC, and NV + EPEC.

On the other hand, the twelve most abundant genera for NoV cohort were *Bifidobacterium*, *Bacteroides*, *Escherichia*, *Veillonella*, *Streptococcus*, *Faecalibacterium*, *Clostridium*, *Ruminococcus*, *Enterococcus* and unclassified genus of *Enterobacteriaceae*, *Veillonellaceae* and *Lachnospiraceae*. *Escherichia*, *Veillonella*, *Streptococcus*, *Faecalibacterium* and an unknown genus belonging to the *Lachnospiraceae* were highly prevalent across all NoV positive samples with an average of 60.8%. [Fig. 1(B)]. NoV + EAEC mixed infections was dominated by high levels of *Streptococcus*, *Escherichia* and *Clostridium* genera. Although similar pattern was observed with NoV + EPEC mixed infections, it was with lesser abundance. It was also interesting to observe that though *Enterococcus* (0.00575) and *Veillonellaceae unclassified* (0.0157) were listed among the top genera by overall relative abundance in NoV cohort, they were found less abundant in NoV + EAEC and NoV + EPEC groups. We observed that *Collinsella* and *Prevotella* which were among the top 12 genera in RV cohort, were not entitled in NoV abundant genera. Similarly, unique genera of *Ruminococcus* and *Enterococcus* were observed in NoV cohort, but were absent in RV cohort. The twelve top bacterial genera and their overall relative abundance per each group including healthy controls and NoV cohort are represented in Supplementary Data S1- Fig. 2. Abundance of specific genera was also noticed in each NoV groups particularly, *Sutterella* (0.0170) (NoV-alone and NoV + EAEC), *Haemophilus* (0.0163) (NoV-alone), *Oscillospira* (0.0377) and *Dorea* (0.022) (NoV + EPEC). In general, we noticed that genus *Bifidobacterium* was less abundant, but prevalent in all the samples of RV and NoV cohorts. On the other hand, genus *Bacteroides* was dominantly abundant in control samples and reduced significantly in RV cohort compared to NoV cohort [Fig. 1(B)].

### Richness, diversity and differential abundance of gut microbiota of AGE children

To further characterize the inter-individual differences between groups (beta-diversity) at the group level, post-hoc test and NMDS were performed. Pairwise differences: post-hoc test of gut microbiome in healthy children and AGE infected children is shown in Table 2. NMDS analysis revealed separation and clustering of samples along NMDS1 axis, whilst species tended to cluster along NMDS2 [Fig. 2(A,B)]. Children with RV infection had significantly scattered clustering compared to the controls (*r*^2^ = 0.074, *p* = 0.013, *pvalBon* = 0.13), with higher significance observed in RV + EAEC (*r*^2^ = 0.146, *p* = 0.009, *pvalBon* = 0.09) and RV + EPEC + EAEC (*r*^2^ = 0.179, *p* = 0.010, *pvalBon* = 0.10) (Fig. 2(A) and Table 2]. However, children with RV + EPEC mixed infections appear to show significant clustering with higher p-value (r2 = 0.101, p = 0.045, pvalBon = 0.45) compared to the controls (Table 2 and Fig. 2). We did not detect obvious significant differences when we compared the diversity of RV alone infected children with their mixed infections groups. On the other hand, significance with higher p-value was observed between RV + EPEC and RV + EPEC + EAEC (*r*^2^ = 0.146, *p* = 0.043, *pvalBon* = 0.43) groups. Children with NoV infection showed microbe clustering compared to healthy samples with higher p-value (*r*^2^ = 0.067, *p* = 0.049, *pvalBon* = 0.29) but high significance was observed in NoV + EAEC (*r*^2^ = 0.165, *p* = 0.009, *pvalBon* = 0.054) [Fig. 2(B)]. Analysis of variance showed no significant association between NoV + EPEC mixed-infection group and controls (*r*^2^ = 0.097, *p* = 0.188, *pvalBon* = 1.12). Overall, the pairwise differences with the control samples indicated that mixed infections with RV cohort had significant associations, specifically RV + EAEC mixed infection (*p* < *0*.*05*). On the other hand, only NoV infection and NoV + EAEC groups showed significant (*p* < *0*.*05*) associations. Moreover, no association was observed with NoV + EPEC group (*p* = *1*.*88*).

**Table 2:** Pairwise differences: post-hoc test of gut microbiome in healthy children and AGE (RV and NoV) infected children.

Group 1	Group 2	Regression (r^2^)	P-value	P-value Bonferroni correction	P-value False Discovery Rate
Control samples	RV-Alone	0.08	0.013^a^	0.13	0.043
Control samples	RV + EAEC	0.15	0.009^a^	0.09	0.09
Control samples	RV + EPEC	0.1	0.045^b^	0.45	0.09
Control samples	RV + EPEC + EAEC	0.18	0.010^a^	0.1	0.05
RV-Alone	RV + EAEC	0.05	0.257^c^	2.57	0.286
RV-Alone	RV + EPEC	0.04	0.334^c^	3.34	0.334
RV-Alone	RV + EPEC + EAEC	0.06	0.141^c^	1.41	0.201
RV + EAEC	RV + EPEC	0.1	0.060^c^	0.6	0.1
RV + EAEC	RV + EPEC + EAEC	0.11	0.174^c^	1.74	0.217
RV + EPEC	RV + EPEC + EAEC	0.15	0.043^b^	0.43	0.107
Control samples	NoV-Alone	0.07	0.049^b^	0.294	0.147
Control samples	NoV + EAEC	0.17	0.009^a^	0.054	0.054
Control samples	NoV + EPEC	0.1	0.188^c^	1.128	0.376
NoV- Alone	NoV + EAEC	0.06	0.207^c^	1.242	0.31
NoV- Alone	NoV + EPEC	0.04	0.570^c^	3.42	0.57
NoV + EAEC	NoV + EPEC	0.08	0.556^c^	3.336	0.667

RV- rotavirus; EAEC-*Enteroaggregative* *E. coli*; EPEC-*Enteropathogenic* *E. coli*; NoV- norovirus.

^a^statistically highly significant between control samples and AGE infection (p < 0.01);

^b^statistically low significant between control samples and AGE infection (p < 0.05);

^c^statistically not significant within AGE (p > 0.05).

**Figure 3:**
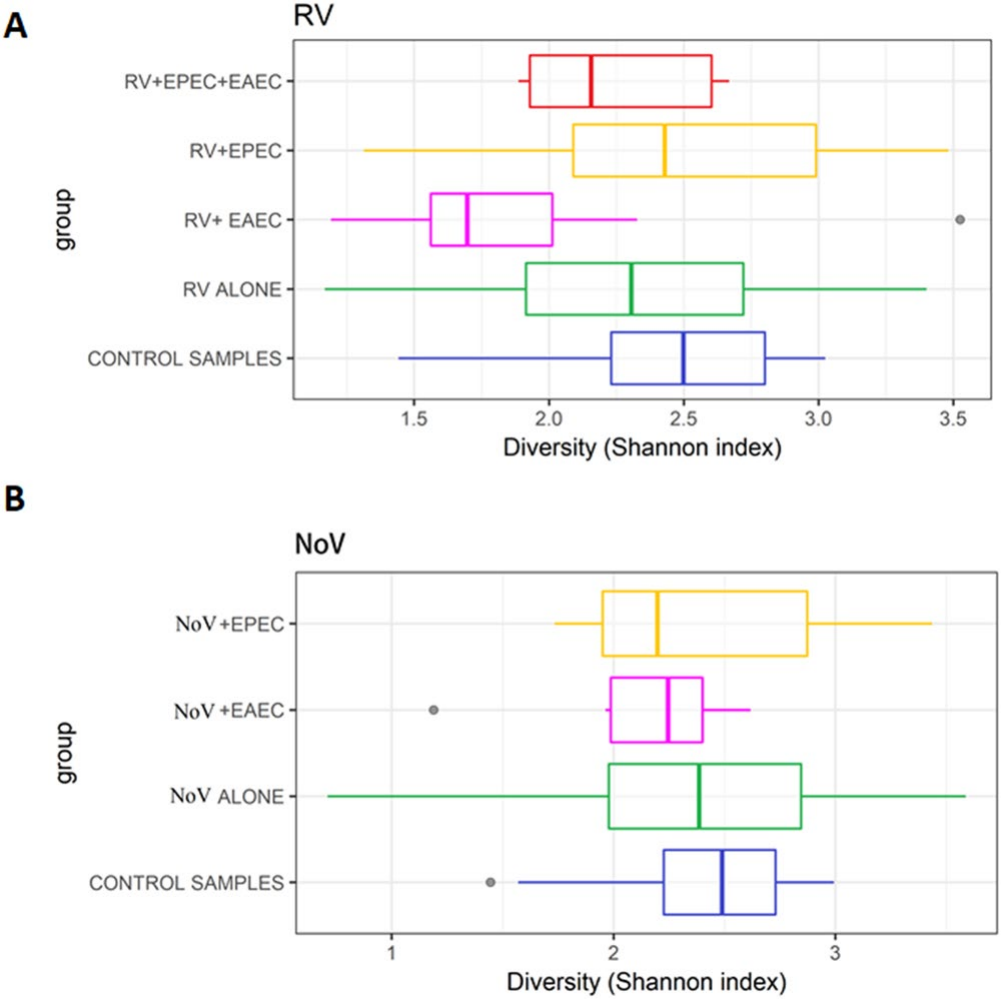
Shannon diversity index of microbiota among healthy controls and infected AGE cohorts. (**A**) Analysis of Shannon diversity index in control samples, RV-alone, RV + EAEC, RV + EPEC, and RV + EPEC + EAEC. (**B**) Analysis of Shannon diversity index in control samples, NV-alone, NV + EAEC, and NV + EPEC.

Shannon diversity index, reported as entropy scores, were calculated for control group and each infected group of children and elucidated in Fig. 3. The entropy score is known to increase as the number of species increases as well the distribution of individuals among the species becomes even. All RV infected groups reported lower entropy scores compared to the control group. Decrease in scores was more significant (p = 0.0024) in the mixed infections with EAEC, especially the RV + EAEC 1.65 (1.6–1.9). On the other hand, entropy scores were less divergent in the NoV groups, recoding the following values: NoV-alone 2.4 (2.0–2.7), NoV + EAEC 2.3 (2.0–2.5), and NoV + EPEC 2.3 (2.0–2.9), [Fig. 3(B)]. In summary, Shannon diversity indices suggested severe alteration in childrens’ gut microbiome communities with RV single and mixed-infections with EAEC.

We observed 14 RV (*p*_adjusted_ < 0.05) and 20 NoV (*p*_adjusted_ < 0.01) differentially abundant OTUs, respectively, in infected children regardless of the bacterial co-infections [Supplementary Data S1- Fig. 3(A,B)]. Each of these OTUs represented 0.025–0.3% of the total abundance in RV cohort, and 0.0001 to 0.2% of the total abundance in NoV cohort. OTUs in RV cohort were associated with diverse range of bacteria including *Roseburia*, *Blautia*, *Parabacteroides faecis*, *Fusobacterium*, *Gemella*, *Moraxella* and *Pullicaecorum* [Supplementary Data S1- Fig. 3(A)]. Only five OTUs were significantly abundant in the RV-alone infected children group (*p*_adjusted_ < 0.05), compared with healthy controls, including *Melaninogenica*, *Blautia*, *Bacteroides*, *Fusobacterium* and *Gemella*. *Melaninogenica* (0.003–0.015%) and *Gemella* (0.007 to 0.0015%) were observed in RV + EAEC whereas *Coprocococu*s (0.005 to 0.010%), *Moraxella* (0.001–0.06%) and *Gemella* (0.002–0.008%) were significantly abundant in RV + EPEC group. On the other hand, seven OTUs were differentially abundant in NoV cohort including *Clostridium Citroniae* (0.001–0.015%), *Haemophilus parainfluenzae* (0.01–0.03%), *Alistipes putredinis* (0.003–0.006%), *Anaerostipes caccae* (0.002–0.04%), *Acinetobacter guillouiae* (0.001–0.006%), *Clostridiales* (0.004–0.012), and *Trachinotus ovatus* (0.01 to 0.03). A comparison between EAEC- and EPEC-infected groups showed differential abundance of *Streptococcus* and *Coprococcus* in both RV + EAEC and NoV + EAEC children, whereas *Coprococcus* and *Melaninogenica* were differentially abundant in both RV + EPEC and NoV + EPEC [Supplementary Data S1- Fig. 3(B)]. Moreover, it was peculiarly noticed that the number of differential abundant pathogenic OTUs were found only with NoV mixed infection compared to RV mixed infected groups.

### Correlation between microbiome composition and clinical manifestations in RV- and NoV- alone infected groups

The correlations of the gut microbiota composition with clinical manifestations were evaluated based on Table 1. The abundance of genus *Clostridium* resulted in increased frequency of diarrhea (6 to 7 times/day) and vomiting (6 times/day) in children with RV positive infection. Further, children with fever had greater richness in *Prevotella* compared to other genera. On the other hand, abundance of genus *Streptococcus* was observed to be associated with increase in diarrhea duration (7–8 days) in children with NoV positive infection.

RV-alone infected children with an increased frequency of diarrhea exhibited greater abundance of Sulfur River 1 (SR1) (50%), Lentisphaerae (50%), Nitrospirae (38%) and Caldiserica (5%), which were absent in NoV- alone infected and healthy children (Supplementary Data S1 – Fig. 4). Dominance of phyla Chlorobi was associated with moderate dehydration in NoV infected children. Microbial profiles and their correlation in phylum level between RV, NoV and healthy pediatric cohorts are shown in Supplementary Data S1 – Fig. 4.

### Microbiome composition and clinical manifestations in mixed infections with EAEC

In the RV + EAEC mixed infection, children with an onset of AGE symptoms for more than 2 days prior to hospitalization exhibited significant abundance and *Escherichia*, and lesser abundance of *Bacteroides*. The abundance of *Bacteroides* was even lesser in NoV + EAEC group compared to RV + EAEC group. There was no abundance of specific genera that correlated with degree of dehydration in RV + EAEC group. However, genera *Escherichia*, *Streptococcus*, *Rumella* and *Clostridium* seemed to be specifically correlated with degree of dehydration in NoV + EAEC group. Analysis at the phylum level for viral-EAEC mixed infections did not show any significant difference in various microbial diversity indices as was observed in RV- and NoV- infections alone (Supplementary Data S1 – Fig. 5).

### Microbiome composition and clinical manifestations in mixed infections with EPEC

In RV + EPEC mixed-infection group, children with high *Streptococcus* abundance had adverse condition of vomiting and diarrhea, reaching approximately 7–8 times per day. Only children infected with *Escherichia* were admitted and hospitalized for more than a day. Similar to our observation with EAEC mixed infection, and in comparison with RV + EPEC infected group, NoV + EPEC mixed-infections resulted in substantial decrease in genus *Bacteroides* as well as *Bifidobacterium* (Supplementary Data S1 – Fig. 6). Importantly, phyla Chlorobi, which was observed absent in RV- alone group, was significantly abundant RV + EPEC mixed infections, but absent in NoV + EPEC. Conversely, phyla Rubiaceae richness was observed only in NoV + EPEC, but absent in RV + EPEC. Furthermore, phyla Nitrospirae was particularly absent in RV- and NoV- mixed infections (Additional File 1).

### Microbiome composition and clinical manifestations in RV vaccinated cohort

The number of children in RV cohort with one or two doses of vaccine represented about 27% and 34.1%, respectively. The most notable difference observed in RVV group with one dose was the abundance of *Clostridium* (1.05E-1) and *E*. *coli* (3.28E-1) compared to two doses vaccination. Microbial composition at phylum level denoted higher levels of Firmicutes in both vaccinated and non-vaccinated children. Two doses vaccination in children resulted in decrease in *Bacteroidetes* compared to one dose or non-vaccinated. When comparing total number of bacteria genera as a whole, RVV vaccinated children either with one or two doses of vaccine, showed less abundance of bacterial genera compared to non-vaccinated children (Supplementary Data S1 - Figs 4–6).

## Discussion

Few studies have evaluated the microbiota of children suffering from enteric viral infection^26^, severe and complicated AGE^24^, and irritable bowel syndrome with diarrhea (IBS-D)^30^. In the current study, we investigated the interplay between viral-bacterial mixed infections in children hospitalized with AGE by evaluating microbiome compositions and clinical outcomes in the studied populations. Our study focused on two major viruses (RV and NoV) as well as two major pathogenic *E*. *coli* (EAEC and EPEC) that are known to cause AGE in children. The number of samples evaluated in this study, especially in the viral infection groups, ensured sufficient statistical power in data analysis and interpretation as compared to previously published studies.

We found that both, taxonomical composition and the diversity of gut microbiome, were disrupted or altered in children infected with single enteric virus, but the disruption was worsened with mixed viral-bacterial infections as compared to healthy controls. Moreover, reduction or richness of specific bacterial genera was linked to increased frequency of diarrhea, vomiting and fever, longer hospitalization and age, but not related to gender of the children (Supplementary Data S1 - Figs 4–6). We observed greater richness in bacterial genera in the RV infected group compared to NoV infected group (additional file 1), which is in contrast to recent study done by Chen *et al*. 2017^24^. According to their study, RV infection resulted in decreased microbiota diversity compared with NoV infection, which has not been reported previously. This difference might be attributed to the NoV samples included in their study. They enrolled children with complicated clinical conditions including convulsion, necrotizing enterocolitis, severe electrolyte imbalance and malnutrition. In addition, all fecal samples collected in that study were at least one week after discontinuation of the antibiotics. This could have severely altered the microbiome diversity in NoV infected children. Moreover, although the overall mean severity was higher in RV infected children in their study, the number of cases evaluated for the comparison was less [RV (n = 5) and NoV (n = 15)]. Additionally, Chen *et al*. study indicated that some of their children were administered antibiotics prior to confirmation of a viral infection, which could have drastically altered the gut microbiome.

In comparison to gut microbiota in healthy controls, our results demonstrated that abundance of taxa *Clostridiaceae* and *Streptococcaceae* in the RV- and NoV-alone infected children had an adverse effect as measured by the increased severity of vomiting and diarrhea. In concordance with our findings, the abundance of these two bacteria was previously reported by Kersten *et al*.,1987^31^ in a study that evaluated pediatric gastroenteritis in primary care and in hospitalized patients. This suggests that the growth of *Clostridiaceae* and *Streptococcaceae* may be promoted by disturbances in normal gut microflora, even in the absence of antibiotics use^32,33^; i.e., induced by gastroenteritis. Direct interaction of *Clostridiaceae* with the epithelial cells results in a cascade of inflammatory processes that can contribute to intestinal diseases such as diarrhea and pseudomembranous colitis^34^. Interestingly, the richness of *Streptococcaceae* was also observed with RV + EAEC infections, but the proportion of its richness declined in both RV + EPEC and RV + EPEC + EAEC mixed infections. Similar observations were noticed with NoV cohort, where *Streptococcaceae* richness was observed in mixed infections with EAEC compared EPEC. The association of *Streptococcaceae* with one *E*. *coli* type (EAEC) infection but not the other (EPEC), regardless of the viral infection, is an interesting observation that requires further assessment. Several other studies have also reported the abundance of the above taxa in other diseases including gastric mucosa-associated microbiota in dyspeptic patients^35^, cystic fibrosis^36^, ulcerative colitis^37^ and chronic rhinosinusitis (CRS)^38^. Recently, efforts to engineer the microbiome to cure *Clostridioides difficile* infections through fecal microbiota transplantation (FMT) has evolved^39^. Being currently in the clinical trial, FMT may elucidate the factors that determine species-species interaction in the gut environment^39^.

With respect to the difference in top genera by overall relative abundance of specific populations, our findings indicate that the populations of *Prevotella* and *Ruminococcus* were significantly increased in RV cohort compared NoV cohort. However, a recent study by Rodríguez-Díaz *et al*., 2017, specifically link the abundance of *Ruminococcus* to lower immunoglobulin A titers against both NoV and RV^40^. The occurrence of these bacteria in RV infected patients could also depend on other factors including host’s secretor status and RV genotype, as described for RV P type (P[6] and P[8]) variants^41^. On the other hand, population based studies have shown that *Prevotella* and *Ruminococcus* possess diagnostic values^42^, where abundance of *Prevotella* has been associated with rural communities^43^, non-Western^44^ and a plant-based diet^45^. These factors were not evaluated in our study and worth investigation in future work.

RVV has been shown to substantially reduce morbidity and mortality among children worldwide. Previous studies suggest that the use of RVV reduce emergency department visits and hospitalizations of children with rotavirus acute gastroenteritis by 70% to 100%. Interestingly, substantial number of RV infected patients in our study had received one (n = 9) or two doses (n = 12) of the RV vaccine. We therefor sought of analyzing the effect of vaccine on microbiome composition in the different groups involved in this study. When comparing total number of bacteria genera as a whole, AGE-affected RV-vaccinated children either with one or two doses of vaccine showed slightly less abundance of bacterial genera compared to non-vaccinated children (Supplementary Data S1 - Figs 4–6). One-dose vaccination with RVV correlated with abundance to *Clostridium* and *E*. *coli*, similar to what has been reported in a recent study by Harris *et al*.^23^. In another study that investigated gut microbiome in RVV-responder and non-responders Ghanaian infants, bacteria belonging to the Bacteroidetes phylum, especially several bacteria related to species from the Bacteroides and Prevotella genera, were significantly associated with a lack of RVV response^22,46^. None of the above studies, including ours, have looked at the RV genotype causing the infection, which could dramatically influence infection severity and consequently gut microbiome. Dietary and nutritional status of infants, history of chronic illness, mixed infections, and other factors could explain the discrepancies between the different studies. Although our study is the only one that evaluated mixed-infection effect on gut microbiota, the low number of patients in the mixed-infection groups did not enable valid statistical comparison between groups regarding vaccine effect.

There was no abundance of specific genera that correlated with degree of dehydration in RV groups. However, genera *Escherichia*, *Streptococcus*, *Rumella* and *Clostridium* seemed to be specifically correlated with degree of dehydration in NoV groups (Supplemental Figs 3–4). Diarrhea and vomiting frequencies were worsened with EPEC mixed infections with both RV and NoV. This is in agreement with previous studies that reported persistent diarrhea in children with viral-EPEC mixed infections^47,48^, where EPEC is known to be the most common cause of acute diarrhea and may also cause persistent diarrhea in children^49^. In our study, most of the children with EPEC mixed infections were admitted and hospitalized for more than a day. Coinfection with EPEC was associated with richness of bacteria *Rothia*, *Leptotrichia*, and *Haemophilus* and poorness of genera *Lactobacillus* and *Prevotella* in both RV and NoV cohorts.

On the other hand, children infected with RV/NoV-EAEC mixed infections showed an onset of AGE symptoms for more than 2–3 days prior to hospitalization and exhibited significant abundance in *Escherichia*. Coinfection with EAEC was associated with richness of bacteria genera *Lachnospiraceae*, *Oscillospira*, *Faecalibacterium and unclassified*. Triple infection with RV + EAEC + EPEC was associated with preponderance of bacterial genera *Collinsella*, *Roseburia*, and *Sutterella*.

Studies detailing viral-bacterial interplays and their effect on microbiome are limited and contradictory. Experimental studies in mice revealed exacerbated disturbance of gut microbiota in viral-bacterial mixed infections compared to single RV or NoV infections^50^. In an earlier study, Hori *et al*. 1996, reported that rotavirus causes more severe gastroenteritis than bacteria or parasites in Ghanaian children^51^. Another study from Bangladesh found that mixed RV and *E*. *coli* infections were similar in severity to infections with *E*. *coli* or RV alone^52^. These results indicate that factors other than the enteric pathogens are responsible for disease manifestations. This partially includes gut microbiome, which is an important player in determining immune response to- and pathogenesis of GIT infection. Our study partially answer some of the questions, while many remained unanswered. A larger follow up study is needed to unveil the contribution of several others factors such as virus genotype, levels of virus replication, mixed infections with other pathogens, genetic makeup of the host and others. Expectedly, *Bacteroidaceae* richness was significantly greater in healthy children as this microflora is known to maintain the physiologic conditions of the colon^53^. On the other hand, reduction of relative numbers of *Bacteroides* was reported in both RV and NoV infections, which was more prominent in the mixed infection with EPEC and EAEC. Reduced numbers of *Bacteroides* have been reported earlier in stools from NoV-infected patients, which seems to have been more deteriorated in viral-bacterial mixed infection as the case in our study^26^. Whether this reduction *Bacteroides* is a consequence of infection, or a predisposition factor that facilitated the infection requires further investigations. Of note, certain *Bacteroidetes* express a form of lipopolysaccharide (LPS) that is structurally and functionally different from the LPS expressed on *E*. *coli*^54^. LPS is a strong immunogenic stimulator, present in the outer membrane of gram-negative bacteria^54^. *Bacteroidetes* LPS has been shown to inhibit innate immune signaling and endotoxin tolerance as compared with that of *E*. *coli*^55^. This suggests that differences in microbiota-derived LPS may modulate aspects of immune education and play a role in determining susceptibility to immune diseases. Alternatively, *Bifidobacteriaceae*, which act as probiotics, seems to increase depending on the severity of the infections. That is, we observed an increase in *Bifidobacteriaceae* richness in the viral-bacterial mixed infections compared to single viral infections (Fig. 1). Studies have proven that the decrease in *Bifidobacteriaceae* impairs the gastroenteritis homeostasis and may result in mucosal inflammation in the GI tract. In addition to *Lactobacillus*, strains belonging to *Bifidobacterium*, are widely used as probiotic bacteria and are included in many functional foods and dietary supplements^56,57^. These probiotics have been reported to suppress diarrhea^58^, exhibit antimicrobial effect^59^, prevent inflammatory bowel disease^60^, and alleviate lactose intolerance and postoperative complications^61,62^. *Bifidobacterium* and *Lactobacilli* have been also shown to inhibit a wide range of pathogens, including *Helicobacter pylori*, *E*. *coli*, *Listeria monocytogenes*, *Salmonella*, and RV^63–66^. Our results suggest that our body has evolved mechanisms to combat GIT disease by promoting probiotic bacteria growth over the aggression of pathogenic ones. Nonetheless, generalizations regarding the potential probiotic health benefits should not be made because they tend to be strain specific.

Despite the differences in the age of children of each subgroup, we believe that this difference was not a confounder and it did not affect the microbiota composition. Although it has been suggested that the composition of bacterial communities evolve towards an adult-like configuration after 1–3 year of age, other studies showed that it continues to change until teenage years^10^. Moreover, having age-matched subgroups is difficult for such studies and here we present the first data set of results describing mixed infections effect on microbiome. We therefore believe that future studies with larger cohorts should count for age, gender, ethnicity, and other factors.

## Conclusion

We evaluated the bacterial composition in viral-bacterial AGE mixed infections in correlation with various clinical severities. Our results revealed that richness of *Bifidobacteriaceae*, which act as probiotics, increased with the severity of the viral-bacterial mixed infections. As expected, significant reduction of relative numbers of *Bacteroides* was characterized in both RV and NoV infection, in which was more prominent in mixed infections with pathogenic *E*. *coli*. Though EAEC mixed infection resulted in significant microbiota alterations compared to virus-alone or virus-EPEC mixed infection, the clinical conditions of the children was worsened with both *E*. *coli* virotypes. This could be attributed to the type of toxins secreted and mode of pathogenesis exerted by each type. However, this study did not look into the virulence factors exerted by each of the *E*. *coli* types on the gut microbial community, which mandates further investigations. Further, augmented number of differential abundant pathogenic OTUs was peculiarly noticed only with NoV mixed infection. Certain pathogenic commensal bacteria express carbohydrates indistinguishable from human histo-blood group antigen (HBGA) and NoV particles bind to these HBGA-expressing bacteria^67^. Interaction of NoV particles with HBGA-expressing bacteria further enhances infection of the host^67,68^. Viral-bacterial interplay in the gut environment is a rather complicated process that involves multiple factors including host genetic makeup, host diet, host immune status, microbiome composition in addition to the infectious agents. Results from this study will facilitate further studies on the interaction between aforementioned factors.

## Methods

### Ethical approvals

This study was approved by the institutional review board (IRB) committees at Hamad Medical Corporation (HMC) and Qatar University (QU). All samples were collected with informed consent signed from the parents/legal guardians under IRB approval from HMC (Approval # 16173/19) and exemption from QU (Approval # QU-IRB605-E/16). Samples were collected with information about age, nationality and clinical data, such as frequency and duration of vomiting, diarrhea, fever, severity of dehydration, and duration of hospitalization. All methods were performed in accordance with the relevant national and international guidelines and regulations.

### Patient enrollment and sample collection

Samples from viral AGE affected children were collected from the Pediatric Emergency Center, Hamad Medical Coorporation, Doha, Qatar. Samples were screened for viral infections (group A RV and NoV), and bacterial infections (EPEC and EAEC) using the FilmArray Gastrointestinal (GI) Panel kit^®^, BioFire Diagnostics, United States. We evaluated the severity of gastroenteritis using the Vesikari score system^69^ with the following definitions: A score of 10–20 indicates severe gastroenteritis, 7 ≤ 10 indicate moderate, and <7 indicate mild illness. A total of 70 young children with confirmed AGE (RV = 18, RV + EAEC = 8, RV + EPEC = 10, RV + EPEC + EAEC = 5, NoV = 17, NoV + EAEC = 7 and NoV + EPEC = 5) along with nine healthy children (negative controls) were enrolled in our study. All the fecal specimens were processed with 10% glycerol, and stored at −80 °C for bacteria preservation.

### Extraction and amplification of 16S rRNA

Total bacterial DNA was extracted using QIAamp UCP Pathogen Mini Kit (Qiagen, Hilden, Germany) according to the manufacturer’s recommendations by using Pathogen Lysis Tubes for efficient lysis of cells of gram negative and gram positive bacteria. Bacterial 16S rRNA genes were PCR-amplified with dual-barcoded primers targeting the V4 region, as per the protocol of Kozich *et al*.^29^. The PCR products were normalized, pooled and quantified before being denatured and spiked with PhiX DNA control, Illumina^®^, Unites States.

### Handling contamination

The potential for contamination was addressed by co-sequencing of the DNA amplified from specimens, and four template-free controls^29^. Two positive controls, consisting of cloned SUP05 DNA, were also included (number of copies = 2*10^6^). Operational Taxonomic Units (OTUs) were considered putative contaminants (and were removed) if their mean abundance in controls reached or exceeded 25% of their mean abundance in specimens.

### Illumina MiSeq sequencing

Amplicons were sequenced with an Illumina MiSeq using the 250-bp paired-end kit (v.2), Illumina^®^, Unites States. The resulted bacterial reads were distributed across the samples, to demonstrate uniform coverage and clustered with the mothur software package (v. 1.39.5)^70^, following the recommended procedure^71^.

### Microbiome data analysis

High quality reads were classified using Greengenes (v. 13_8) as the reference database. Alpha diversity was estimated with the Shannon index on raw OTU abundance tables after filtering out contaminants by using the mothur version of the Bayesian classifier^71^. Positive correlation between relative abundance and prevalence was calculated using Spearman’s correlation coefficient. To estimate beta diversity across samples, we excluded OTUs occurring in fewer than 10% of the samples with a count of less than three and computed Bray-Curtis indices^72^. We visualized beta diversity, emphasizing differences across samples, using non-metric multidimensional scaling (NMDS) ordination. Pairwise differences: post-hoc test was estimated to characterize the inter-individual differences between groups (beta-diversity) at the group level. Variation in community structure was assessed with permutational multivariate analyses of variance (PERMANOVA) with treatment group as the main fixed factor and using 4,999 permutations for significance testing. All analyses were conducted in the R environment. To determine which OTUs were driving the differences in microbial composition, we analyzed negative binomial Generalized Linear Models on each OTU with the DESeq. 2 R package (v. 1.19.1)^73^. Hypothesis testing was achieved with likelihood ratio tests. *P*-values were adjusted using the Bonferroni correction (*p*-values threshold = 0.05) and Benjamini-Hochberg procedure^74^.

## Supplementary information

Figures

Dataset 1

## Data Availability

All the necessary data and materials are included in as supporting data and additional files.
