# Supplementary material for: Mixed Viral-Bacterial Infections and Their Effects on Gut Microbiota and Clinical Illnesses in Children
Source: Sci Rep. 2019 Jan 29;9:865. doi: 10.1038/s41598-018-37162-w (PMC6351549; doi:10.1038/s41598-018-37162-w)
Supplement: Supplementary file 1 — Figures [file 41598_2018_37162_MOESM1_ESM.pdf]

Mixed Viral-Bacterial Infections and Their Effects on Gut Microbiota and Clinical Illnesses in Children

Shilu Mathew[1], Maria K. Smatti[1], Khalid Al Ansari[2], Gheyath K. Nasrallah[1, 3], Asmaa A. Al Thani[1,3], Hadi M. Yassine[1,3]*

[1] Biomedical Research Center, Qatar University, Doha, Qatar 2713
[2] Pediatric Emergency Center, Hamad Medical Corporation, Doha, Qatar 3050
[3] Department of Biomedical Science, College of Health Science, Qatar University, Doha, Qatar 2713

Supplementary figure 1A

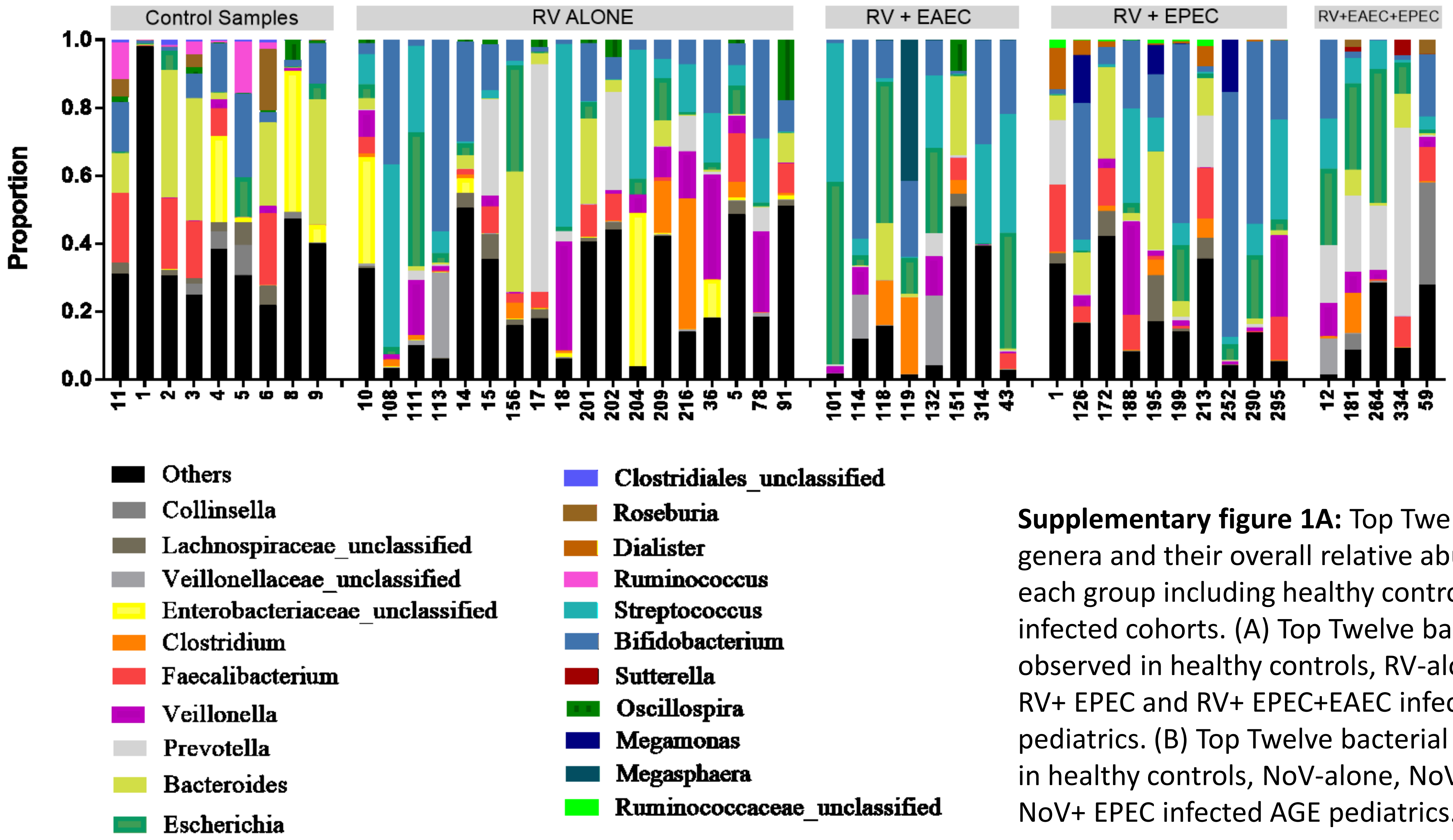

Supplementary figure 1A: Top Twelve bacterial genera and their overall relative abundance per each group including healthy controls, RV and NoV infected cohorts. (A) Top Twelve bacterial genera observed in healthy controls, RV-alone, RV+ EAEC, RV+ EPEC and RV+ EPEC+EAEC infected AGE pediatrics. (B) Top Twelve bacterial genera observed in healthy controls, NoV-alone, NoV+ EAEC, and NoV+ EPEC infected AGE pediatrics.

Supplementary figure 1B

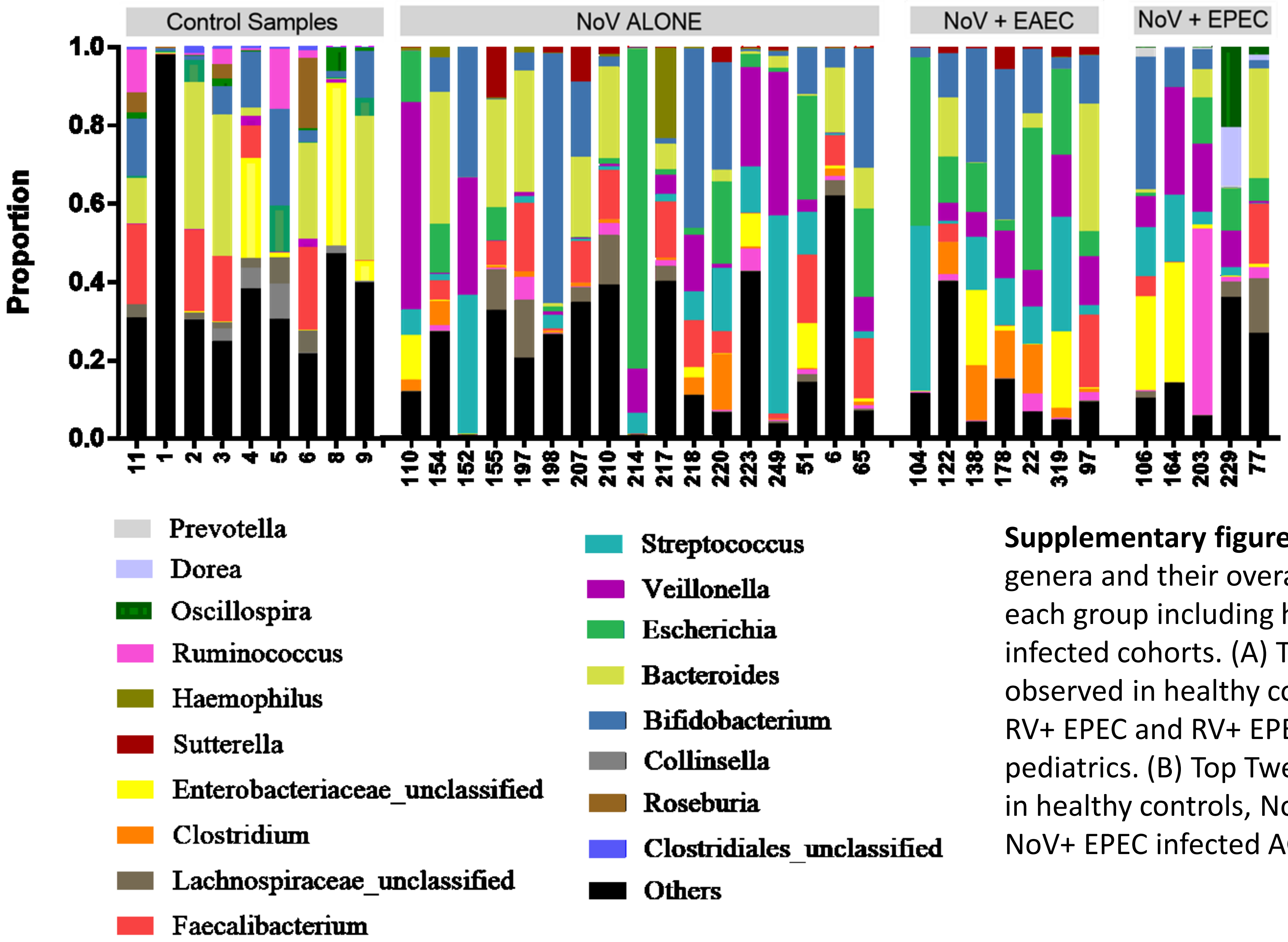

Supplementary figure 1B: Top Twelve bacterial genera and their overall relative abundance per each group including healthy controls, RV and NoV infected cohorts. (A) Top Twelve bacterial genera observed in healthy controls, RV-alone, RV+ EAEC, RV+ EPEC and RV+ EPEC+EAEC infected AGE pediatrics. (B) Top Twelve bacterial genera observed in healthy controls, NoV-alone, NoV+ EAEC, and NoV+ EPEC infected AGE pediatrics.

Supplementary figure 2

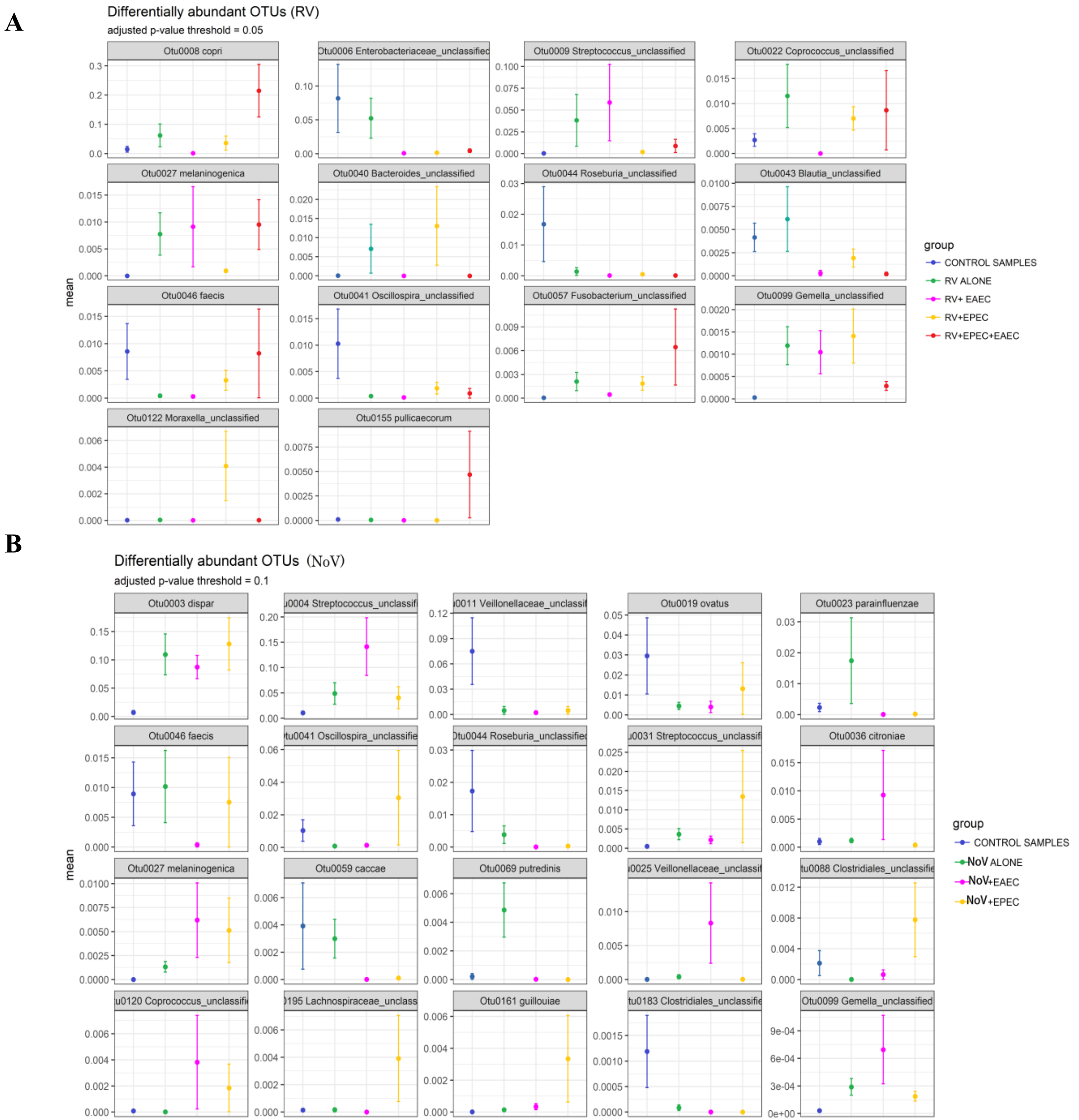

Supplementary figure 2: Statistical analysis of differentially abundant OTUs in RV and NoV cohorts. (A) Differentially abundant OTUs present in control samples and RV cohorts (adjusted p-value threshold=0.05). (B) Differentially abundant OTUs present in control samples and NoV cohorts (adjusted p-value threshold=0.1).

Supplementary figure 3

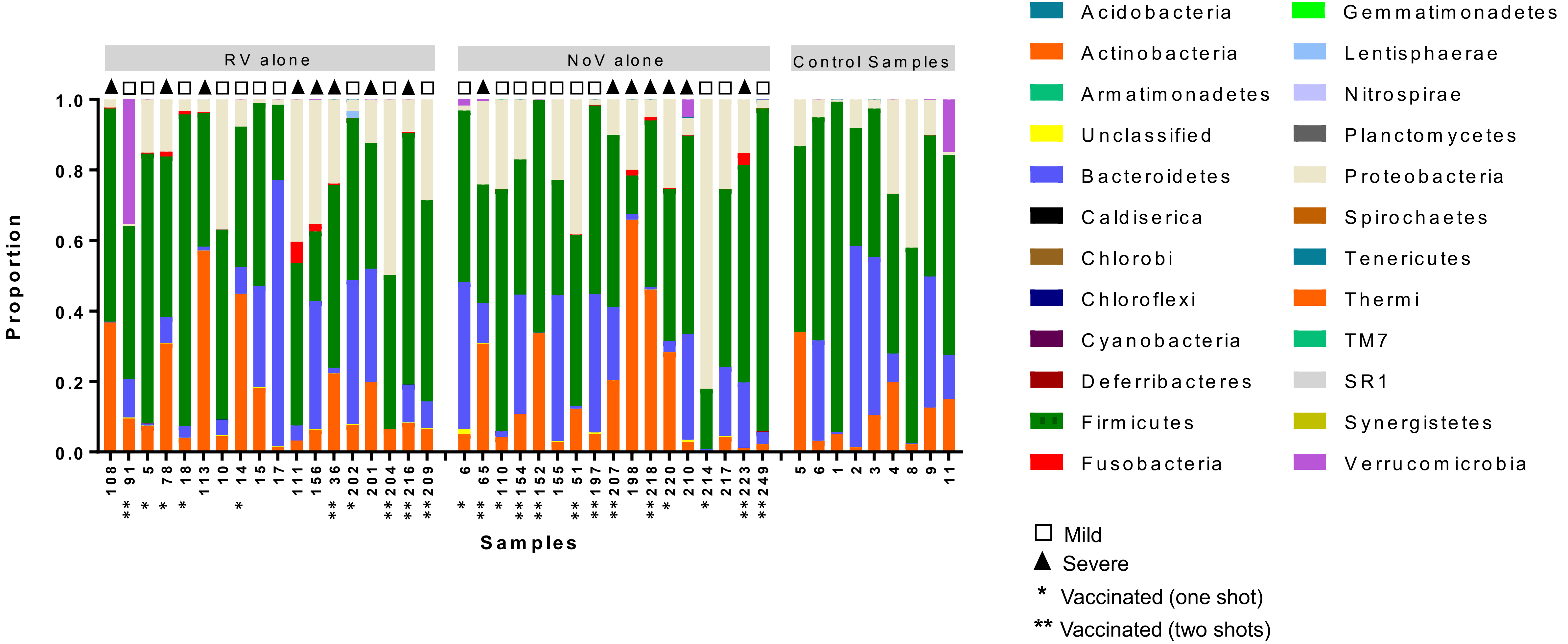

Supplementary figure 3. Microbial profiles represented in phylum level for each group with RVV vaccination and severity of the disease: RV infected, NoV infected, and healthy controls.

Supplementary figure 4

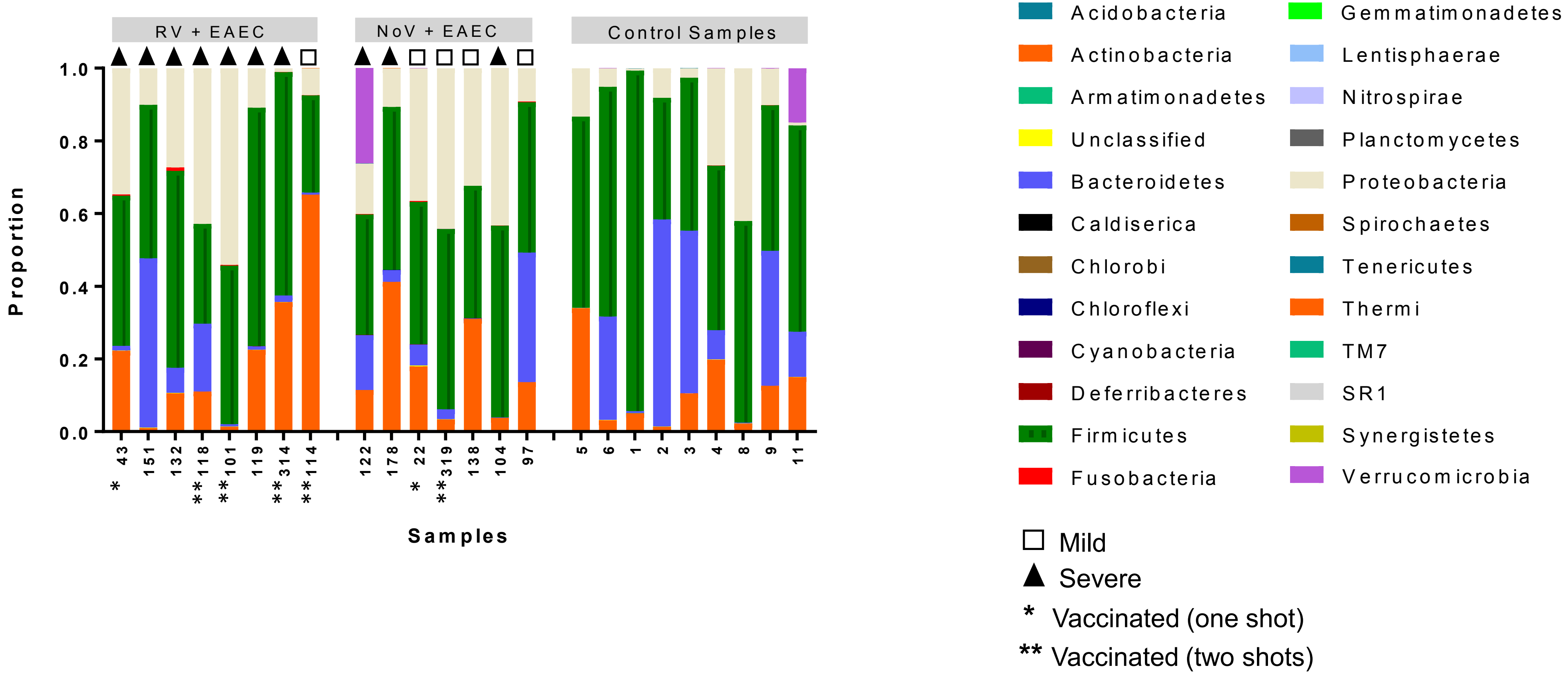

Supplementary figure 4. Microbial profiles represented in phylum level for each group with RVV vaccination and severity of the disease: RV + EAEC, NoV + EAEC, and healthy controls.

Supplementary figure 5

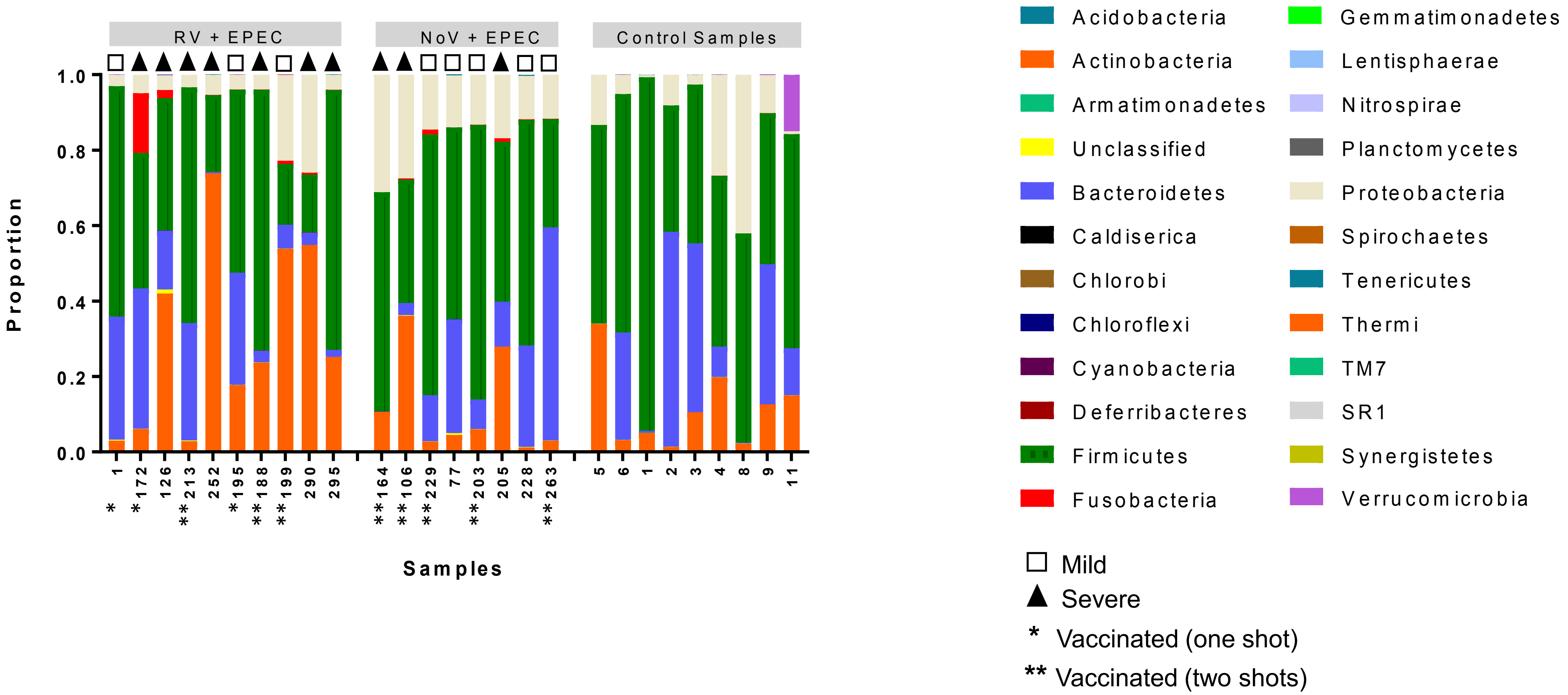

Supplementary figure 5. Microbial profiles represented in phylum level for each group with RVV vaccination and severity of the disease: RV + EPEC, NoV + EPEC, and healthy controls.
